# Practical Points

**Published:** 1905-02-11

**Authors:** 


### PRACTICAL POINTS.

### THE AUTHORITY OF HONORARY SECRETARIES.

A Correspondent writes:—I have been matron of this hospital for six months—the members of the committee are all entirely new to hospital administration—and I should be glad if you would kindly advise me on one or two points.

Is it usual for the honorary secretary (there is a paid one) to be constantly about the building interfering with the working of the household staff? Also, when the matron orders groceries, is it the rule for the order to be countersigned by one of the committee? The orders are all written on printed forms, without which no order may be given.

[Each hospital should have an administrative head. In some of the smaller hospitals the honorary secretary is the chief administrator, and by the rules he is responsible for the management when the committee is not sitting. Our correspondent does not send a copy of the last report or of the rules, and we cannot deal with the particular points raised, in the absence of these documents.—Ed. The Hospital.]

